# Reductive soil disinfestation by mixing carbon nanotubes and mushroom residues to mitigate the continuous cropping obstacles for *Lilium Brownii*

**DOI:** 10.1007/s44297-024-00023-2

**Published:** 2024-03-15

**Authors:** Ding‑Di Tu, Rong Song, Bei Yan, Jin-Feng Dai, Hua Fang, Qian-Qi Zheng, Yi Gu, Xiao-Lan Shao, Hong Chen, Meng-Long Li, Kai-Lin Liu

**Affiliations:** 1 College of Plant Protection, Hunan Agricultural University, Changsha, 410128 PR China; 2 Institute of Agricultural Environment and Ecology, Hunan Academy of Agricultural Sciences, Changsha, 410125 China; 3 Hunan Provincial Institute of Product and Goods Quality Inspection, Changsha, 410007 China; 4 Key Laboratory of Molecular Biology of Crop Pathogens and Insects, Ministry of Agriculture, Zhejiang Provincial Key Laboratory of Biology of Crop Pathogens and Insects, Institute of Pesticide and Environmental Toxicology, College of Agriculture and Biotechnology, Zhejiang University, Hangzhou, 310058 China

**Keywords:** Lily, Continuous cropping obstacle, Soil physicochemical properties, Microbial community structural

## Abstract

Reductive soil disinfestation can effectively alleviate continuous cropping obstacles. The effects of mixing mushroom residues and carbon nanotubes on the structure and function of soil microbial communities and self-toxic substances in *Lilium brownii* var are still unclear. We evaluated the nature of different soil treatments on soil physicochemical properties, microbial community structural response, and the impact of self-toxic substances. Compared with the compound fertilizer treatment, both organic fertilizer and mushroom residues treatments improved soil pH to 7.06 and 6.57, respectively. Carbon nanotubes treatment increased the relative abundance of Acidobacteriota by approximately 3.96%, although it could not significantly affect the soil pH. The mushroom residues flooded cover film treatment increased the soil pH to 6.75, replenished soil elements, and increased the relative abundance of Verrucomicrobiota and Campylobacterota by 1.62% and 1.79%, respectively. Carbon nanotubes and mushroom residues flooded cover film treatment significantly increased the diversity of the bacterial community. The relative abundances of Acidobacteriota, Verrucomicrobiota, Actinobacteria, and Chloroflexi increased by 2.4%, 1.39%, 0.80%, and 0.53%, respectively. Additionally, the minimum number of self-toxic substances was detected through GC–MS analysis. The results of this study showed that mushroom residues flooded cover film treatment could directly improve the soil microbial community structure by changing the physicochemical properties of the soil, and mixed carbon nanotubes further adsorbed the self-toxic substances. The species composition of fungal communities in the soil had been altered in a way by the different treatments, but the fungal diversity didn't change significantly. This research provides a theoretical basis for revealing the mechanism of continuous cropping obstacles in lily and offering better guidance for sustainable development in lily.

**Supplementary Information:**

The online version contains supplementary material available at 10.1007/s44297-024-00023-2.

## Introduction

In recent years, the application scope of lilies has continuously expanded, with sustained growth in both domestic market demand and export volume. The planting area of continuous cropping lilies continues to increase [1]. This is one of the key factors affecting the sustainable development of Lily production and its related industries. In addition to the deterioration of soil physics, soil microecological imbalance is also an important reason [2]. Therefore, according to the continuous obstacle to forming a mechanism, researchers adopted a series of measures to alleviate the continuous obstacles of lily.

The previous research on the mechanism and regulation technology of the continuous cropping obstacles in the planting industry, proposed methods such as rotating stubbornness, intercropping, soil microbial bacteria, and strong restoration of soil disinfection, etc., to promote the growth of continuous plants. The restoration of soil disinfection method can manufacture a strong restored environment in the short term to quickly repair the continuous land soil to achieve the purpose of eliminating the obstacles, killing the soil disease, and improving the production of crops [3]. In addition, a strong restoration soil disinfection method can improve the soil pH and improve the structure of microorganisms [4]. Reductive soil disinfestation is usually crushed plant straw, which has a small effect on increasing soil microorganisms. However, mushroom residues amendment could increase the growth of cucumber seedlings and the abundance of beneficial microbes in cucumber continuous cropping soil [5]. Soil microorganism is the most active part of the soil [6], leading to the circulation of nutrients, the decomposition of organic matter, and the maintenance of soil fertility [7]. Changes in the microbial community structure reflect the quality and activity of the soil to a certain extent [8]. Carbon nanotubes have great application potential in terms of adsorption, catalytic, and soil remediation because of their special one-dimensional pore nanostructure [9]. Therefore, we speculate that the flooding cover of mushroom residues can improve the structure of the soil microbial community and reduce the problem of obstacles. Additionlly, hybrid multi-wall carbon nanotubes can further absorb self-toxic substances, thereby strengthening the relief of lilies.

To validate this hypothesis, we selected soil from lily fields cultivated for two years as the research subject. We created anaerobic conditions by inundating the soil with mushroom residues and covering it with a film to eliminate pathogenic bacteria while promoting the growth of beneficial microbes. Additionally, carbon nanotubes were introduced to adsorb self-toxic substances, thereby enhancing soil quality. Six different soil treatments were implemented, and the physicochemical properties of the soils subjected to each treatment were analyzed. Illumina NovaSeq high-throughput sequencing technology was employed to investigate the response of soil microbial communities to various treatments. Furthermore, gas chromatography-mass spectrometry (GC–MS) was utilized to analyze the components of the self-toxic substances. These findings provide theoretical support and technical guidance for the sustainable development of lilies.

## Materials and methods

### Study location, experimental design, and soil

The experimental greenhouse is located at the Hunan Institute of Agricultural Environment and Ecology. The test materials were soil from two years of continuous cropping, carbon nanotubes (multi-walled carbon nanotubes) and ternary compound fertilizer of potassium sulfate (N: P: K = 15:15:15). The experiment was set up with six treatments, that is, soil + 0.2% compound fertilizer (CF), soil + 2% organic fertilizer (OF), soil + 0.2% compound fertilizer + 0.5% carbon nanotubes (CF_C), soil + 0.2% compound fertilizer + 3% mushroom residues (CF_M), soil + 0.2% compound fertilizer + 3% mushroom residues flooded cover film (CF_MF) and soil + 0.2% compound fertilizer + 0.5% carbon nanotubes + 3% mushroom residues flood cover film (CF_C_MF). In December 2021, two healthy and undamaged *Lilium brownii* var. *viridulum* scales were selected and sown in plastic pots containing 4.4 kg of soil, with 3 mm buried pieces and a soil thickness of around 15 mm. Among them, the flooding treatment directly immersed the entire plastic basin in the water to segregate the air for 7 d, and then the film was removed and left to dry for 10 days. After sowing for 3 to 4 weeks, collect soil samples. When sampled, two sampling points are arranged in each pot to remove debris covered with plants covered on the surface. After the soil is mixed, it is packed in a self-sealing bag and brought back to the laboratory. Remove impurities such as plant root systems. After 2 mm sieves, one part is used for soil physical and chemical analysis, and the other part is placed in a -80℃ refrigerator for soil DNA extraction.

### Soil physical and chemical analysis

Total nitrogen (TN) was measured by the Kjeldahl method; total phosphorus (TP) was measured by NaOH melting molybdenum antimony anti-colorimetry; total potassium (TK) was measured by a NaOH melting flame photometer; soil organic matter (SOM) was measured by the potassium dichromate volumetric method; soil available phosphorus (AP) was measured by NaHCO_3_ leaching molybdenum-antimony anti-absorption spectrophotometry; soil available potassium (AK) was measured by ammonium acetate extraction and flame photometry; soil pH in water was measured at a soil/water ratio of 2:5 (w: v); and soil EC (soil electrical conductivity, water: soil = 10:1) was measured with a conductivity meter [10–12].

### Soil DNA extraction, PCR amplification, and high-throughput sequencing

The genomic DNA of the samples was extracted by the CTAB or SDS method, and then the purity and concentration of the DNA were detected by agarose gel electrophoresis, An appropriate amount of sample DNA was taken into a centrifuge tube, and the samples were diluted with sterile water to 1 ng/μL. The diluted genomic DNA was used as a template for PCR, and according to the selection of the sequencing region, the primers with Barcode, Phusion® High-Fidelity PCR Master Mix with GC Buffer from New England Biolabs, and high-fidelity enzymes were used to ensure the amplification efficiency and accuracy. England Biolabs' Phusion® High-Fidelity PCR Master Mix with GC Buffer and high-efficiency high-fidelity enzymes were used for PCR to ensure amplification efficiency and accuracy. The 16S V4 region primers (515F and 806R) and ITS1 region primers (ITS5-1737F and ITS2-2043R) were utilized for the identification of bacterial and fungal diversity, respectively. The PCR products were detected by electrophoresis using a 2% agarose gel; PCR products that passed the test were purified by magnetic beads, quantified by enzyme labeling, and mixed in equal amounts according to the concentration of PCR products, and the PCR products were detected by electrophoresis using a 2% agarose gel after mixing sufficiently, and the products were recovered by using the Gel Recovery Kit provided by Qiagen for the target bands. The library was constructed using a TruSeq® DNA PCR-Free Sample Preparation Kit. The constructed library was quantified by Qubit and Q-PCR, and after the library was qualified, it was sequenced using NovaSeq6000.

### High‑throughput sequencing data analysis

According to the Barcode sequence and PCR amplification primer sequences from the downstream data to split the data of each sample, truncate the Barcode and primer sequences and then use FLASH to splice the reads of each sample, and the spliced sequences obtained are the raw tags; the spliced Raw Tags are processed by referring to the Tags quality control of Qiime. The spliced Raw Tags are processed concerning Qiime's Tags quality control process to obtain high-quality Tags data. The Tags obtained after the above processing need to be processed to remove chimeric sequences, Tags sequences are compared with the species annotation database to detect chimeric sequences, and ultimately, chimeric sequences are removed to obtain the effective tags.

The Uparse algorithm is used to cluster all Effective Tags of all samples, and by default, the sequences are clustered into OTUs (Operational Taxonomic Units) with 97% consistency (Identity), At the same time, representative sequences of OTUs will be selected, and based on the principle of its algorithm, the screening is the sequences appearing in the OTUs with the According to the principle of its algorithm, the sequence with the highest frequency of occurrence among OTUs is selected as the representative sequence of OTUs. Species annotation was performed on the OTUs sequences, and the species annotation analysis was performed using the blast method in Qiime software (version 1.9.1) with the Unite (v8.2) database and the alpha diversity statistics of each sample were calculated, including indices such as Chao1, ACE and Shannon. Principal Coordinate Analysis (PCoA) was performed based on Weighted Unifrac distances. All sequences can be obtained from the NCBI Sequence Read Archive (SRA) database, with accession numbers PRJNA1068330 (16S) and PRJNA1068504 (ITS).

### Detection of self-toxic substances

Take 10 g of soil samples, add 50 mL of sterile water, oscillation, soaking, centrifuge, and obtain the soil leachate. Use analysis to extract 50 mL soaking liquid solution with pure dichloromethane, and the evaporator is concentrated to dry at 30℃, adds 2 mL dichloromethane to dissolve, passed through a 0.22 μm filter, and then is used for GC–MS analysis.

GC–MS detection condition: electronic bombardment sources, bombardment voltage of 70 eV, ion source temperature of 230℃, inlet temperature of 260℃, pillar temperature of 50℃ (keep 5 min), heating up at 5℃/min program to 250℃ (Keep 10 min). Scanning range M/Z35-550 amu, HP-5MS column, loading gas is He, flow of 1 mL/min, no diversion of samples, and the amount of input is 1uL. Utilize the NIST17 database for comparison and conduct manual analysis, retaining compounds with a similarity of ≥ 80%. Identify the chemical structures and names of each component.

### Data analysis

Perform one-way analysis of variance (ANOVA) on all data using SPSS 25 software, and calculate the differences between treatments using LSD at a probability level of 0.05. Figures were generated using Origin 2018.

## Results

### Effects of different treatments on soil physicochemical properties

**Fig. 1:**
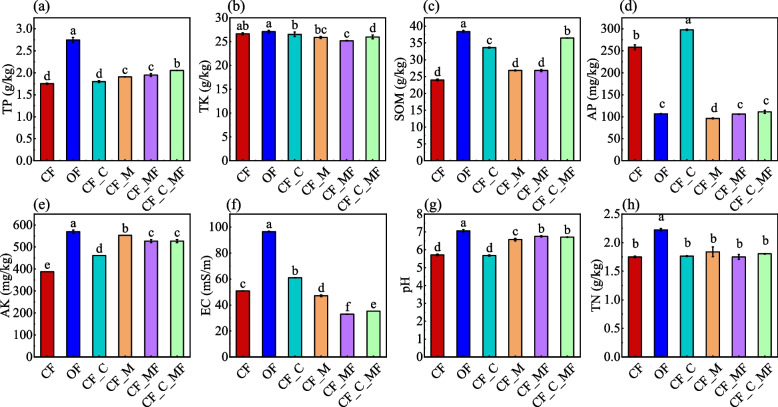
Effects of each treatment on soil physicochemical properties. Different letters indicate significant differences between the groups (*P* < 0.05)

Different soil treatments will have a certain impact on the nature of soil physical and chemical. The nature of the soil processing of each treatment is shown in Fig. 1, the soil pH of CF treatment is 5.71, and the soil is acidic, compared with it, the OF treatment effectively relieves soil acidification, with a pH of 7.06. Similarly, the CF_M, CF_MF, and CF_C_MF treatments can also improve soil pH, measuring 6.57, 6.75, and 6.71, respectively. However, no significant changes have been seen in CF_C treatment; organic matter is usually considered an important indicator of soil quality [13], compared with CF treatment, OF, CF_C, and CF_C_MF treatment have improved; TN and TP have the highest content in OF treatment; AP was significantly higher in CF_C treatment than other treatments, followed by CF treatment; AK is the highest in OF treatment, and the lowest in CF treatment; compared with CF treatment, the conductivity of CF_M, CF_MF, and CF_C_MF treatments significantly decreased, while OF and CF_C treatments were the opposite.

### Effects of reductive soil disinfestation on the growth of lilies

Measure the height of lily plants on May 1, 2022. As shown in Table 1, the seedling height in descending order is CF_C_MF > CF_MF > CF_C > CF > OF > CF_M, and CF_MF and CF_C_MF have significantly higher plant heights than other treatments.

**Table 1:** Effects of reductive soil disinfestation on the growth of lilies

Treatment	Plant height(cm)
CF	10.39 ± 14.46 b
OF	9.67 ± 9.78 b
CF_C	10.85 ± 13.04 b
CF_M	8.42 ± 10.21 b
CF_MF	19.86 ± 18.05 a
CF_C_MF	20.30 ± 27.17 a

The same letter in the same column indicates no significant difference between treatments, while different letters indicate significant differences between treatments (*P* < 0.05)

### Diversity analysis of soil microbial communities

#### The bacterial and fungal communities in lily soil α analysis

**Fig. 2:**
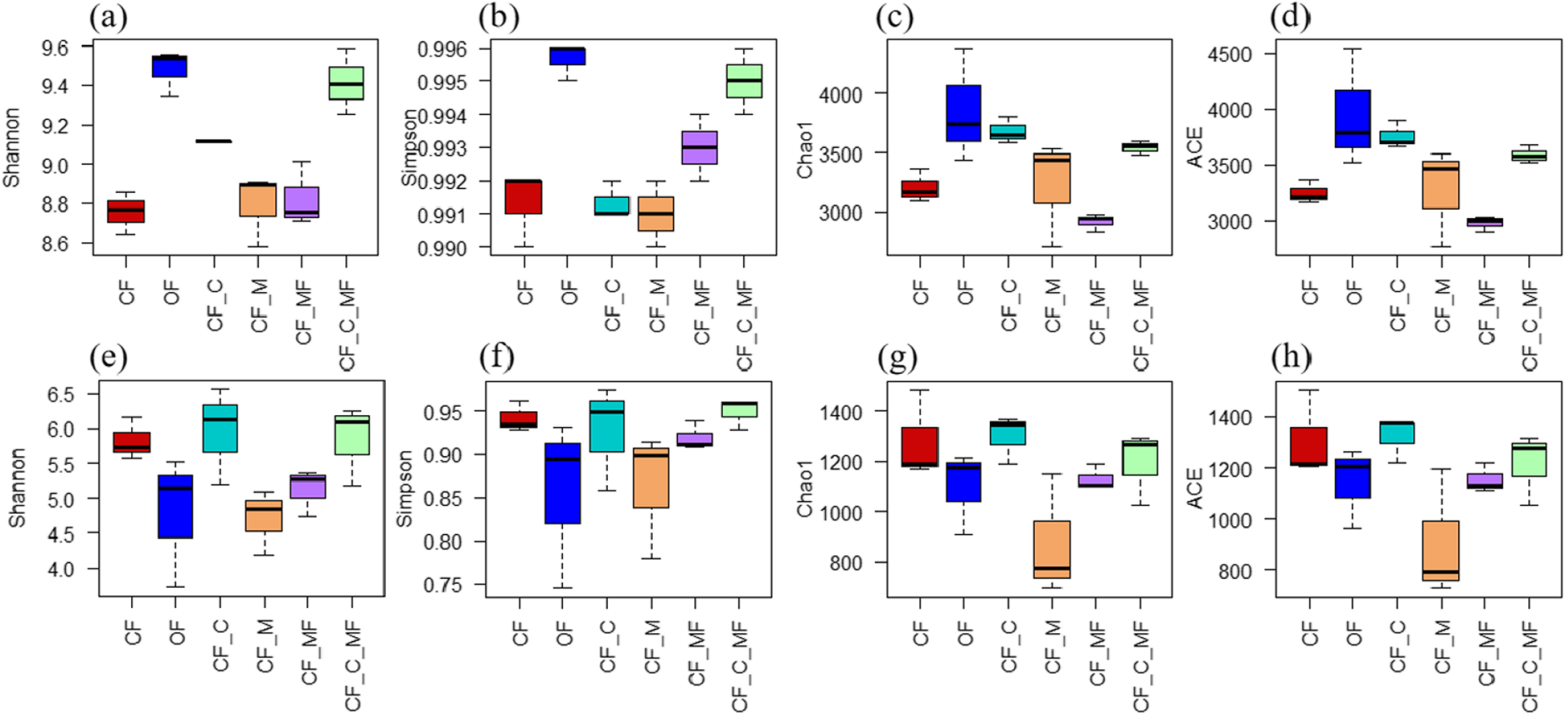
The bacterial and fungal communities in lily soil α Diversity. **a** The bacterial Shannon diversity index. **b** The bacterial Simpson index. **c** The bacterial Chao 1 index. **d** The bacterial ACE index. **e** The fungal Shannon diversity index. **f** The fungal Simpson index. **g** The fungal Chao 1 index. **h** The fungal ACE index

From the Alpha diversity of the soil bacterial and fungal communities under the different soil treatments (Fig. 2), it can be seen that compared with CF, the diversity and richness of OF and CF_C_MF species in the bacterial community are significantly increased; There is no significant difference in the species diversity and richness of the fungal communities.

#### The bacterial and fungal communities in lily soil β analysis

**Fig. 3:**
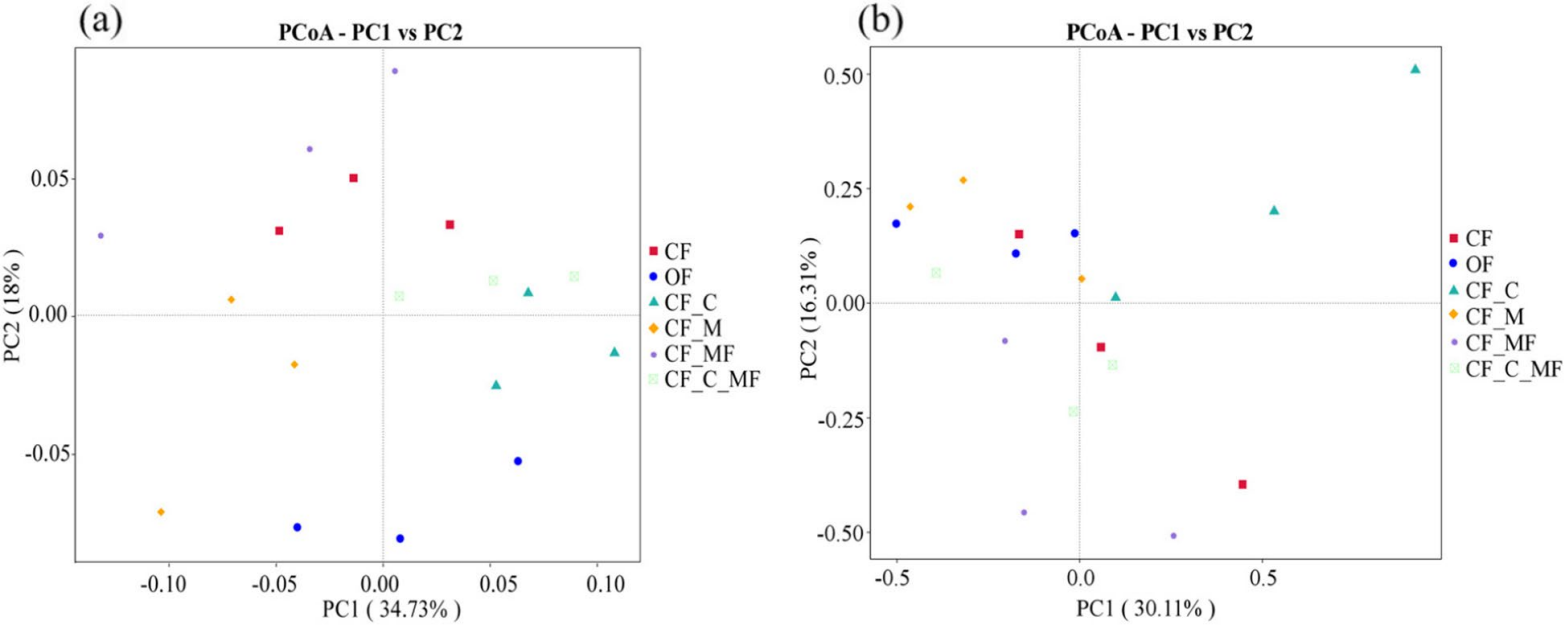
Soil bacteria (**a**), fungal communities (**b**) OTU β Diversity

Principal Co-ordinate Analysis (PCoA) is a dimensionality reduction analysis based on a distance matrix, which evaluates the explanatory power of each coordinate axis on the overall differences in microbial community structure as a percentage. Figure 3 shows that the contribution rates of the PC1 axis and PC2 axis of bacteria are 34.73% and 18%, respectively, with a cumulative contribution rate of 52.73%. Meanwhile, PCoA analysis showed that there was a certain distance between the six soil samples, indicating that the different soil treatments changed the structure of soil bacterial communities.

The contribution rates of the PC1 axis and PC2 axis of fungi are 30.11% and 16.31%, respectively, with a cumulative contribution rate of 46.42%. PCoA analysis showed that the distances between the six soil samples were relatively similar, indicating that the different soil treatments had little impact on the structure of soil fungal communities.

### Analysis of the soil microbial community structure

**Fig. 4:**
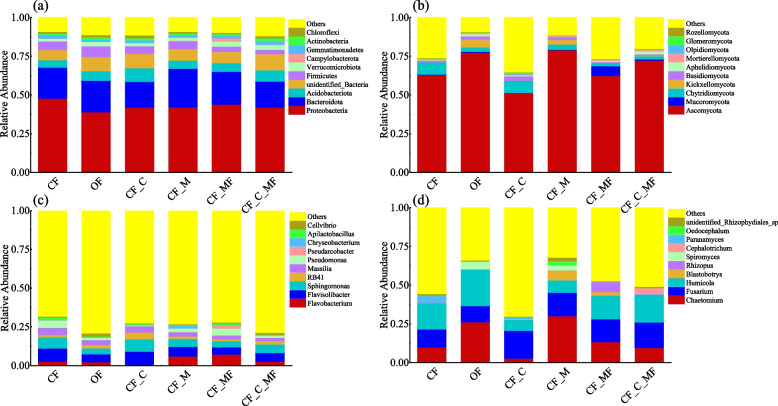
Soil microorganisms of the six samples. **a** Relative abundance at the bacterial phylum level. **b** Relative abundance at the fungal phylum level. **c** Relative abundance at the genus level of bacteria. **d** Relative abundance at the fungal genus level

The composition and relative abundance of the lily soil bacteria and fungal communities are shown in Fig. 4. One color represents a species. The top 10 dominant bacteria and fungi are ranked at the level of phylum. As can be seen in Fig. 4a, at the phylum level, the common dominant phylum of lily soil bacteria in the six treatments were Proteobacteria, Bacteroidota, Acidobacteriota, unidentified_Bacteria, Firmicutes, Verrucomicrobiota, and Gemmatimonadetes. Among them, the proportion of Proteobacteria and Bacteroidota is the highest, accounting for about 60% of the total bacterial abundance. Compared with CF treatment, the relative abundance of the other five treatments of Proteobacteria decreases by more than 1%, Gemmatimonadetes have not changed much in CF_C_MF treatment, and the other four treatment have been reduced; The relative abundance of Firmicutes increased in the OF treatment and was reversed in the CF_MF and CF_C_MF treatments; Verrucomicrobiota increased in OF, CF_MF, and CF_C_MF treatments. Campylobacterota was the dominant phylum in the CF_MF treatment with about 1.99%. Actinobacteria was the dominant phylum in OF, CF_C, and CF_C_MF treatments with 1.52%, 1.39%, and 1.75%, respectively. Chloroflexi was the dominant phylum in OF, CF_C, CF_M, and CF_C_MF treatments with 1.44%, 1.83%, 1.08%, and 1.43%, respectively.

As shown in Fig. 4b, at the phylum level, the common dominant phylum of lily soil fungal in the six treatments were Ascomycota and Chytridiomycota, which accounted for about 50% of the total fungal abundance. Mucoromycota was the dominant phylum in CF_MF treatment with about 6.44%. Kickxellomycota was the dominant phylum in OF and CF_M treatment with about 5.00% and 3.14%. Basidiomycota was the dominant phylum in OF, CF_C, CF_M and CF_C_MF treatments with about 1.98%, 2.78%, 1.79% and 1.06%. Aphelidiomycota was the dominant phylum in OF and CF_C_MF treatments with about 1.53% and 1.95%. Mortierellomycota was the dominant phylum in CF_C_MF treatment with about 1.07%.

The top 10 dominant bacteria and fungi are ranked at the level of genus. As can be seen in Fig. 4c, at the genus level, the six treatments of lily soil bacteria shared the dominant genera of *Flavisolibacter*, *Sphingomonas*, *RB41*, and *Massilia*. *Flavobacterium* and *Pseudomonas* were the dominant genera shared by CF, OF, CF_M, CF_MF, and CF_C_MF treatments. *Pseudarcobacter* was the dominant genus in CF_MF treatment with 2.00%. *Chryseobacterium* was the dominant genus in CF_M treatment with 1.65%. *Apilactobacillus* was the dominant genus in CF and CF_MF treatments with 1.41% and 1.01%, respectively. *Cellvibrio* was the dominant genus in OF and CF_C_MF treatments with 1.91% and 1.11%, respectively.

As shown in Fig. 4d, the fungi share the dominant genera of *Chaetomium*, *Fusarium*, and *Humicola*. *Blastobotrys* had the highest relative abundances of 6.37% and 2.19% in CF_M and CF_MF treatments. *Rhizopus* was the dominant genus in CF_MF treatment with 6.35%. *Spiromyces* was the dominant genus in OF and CF_M treatments with 5.00% and 3.14%. *Cephalotrichum* was the dominant genus in CF_C_MF treatment with 3.59%. *Paramyces* was the dominant genus in CF and CF_C treatment with 4.45% and 1.40%. *Oedocephalum* was the dominant genus in CF_M treatment with 2.46% and it was not detected in CF_MF and CF_C_MF treatments. Unidentified_*Rhizophydiales*_sp was the dominant genus in CF and CF_M treatments with 1.01% and 2.24%.

*Penicillium* and *Acremonium* are the two main pathogens that cause basal and soft rots in lilies. Illumina NovaSeq sequencing results, as shown in Supplementary Information Fig. S1, showed that the patterns of occurrence of the main two pathogenic fungal of lily were different among the six treatments, and there were significant differences in the proportions (percentages) of the two pathogenic fungal to all fungal. *Penicillium* increased significantly in CF and CF_C treatment, and *Acremonium* increased significantly in OF treatment.

### Effects of different treatments on intergroup variability at the phylum level and genus level of the dominant bacterial flora of continuous lily soils

**Fig. 5:**
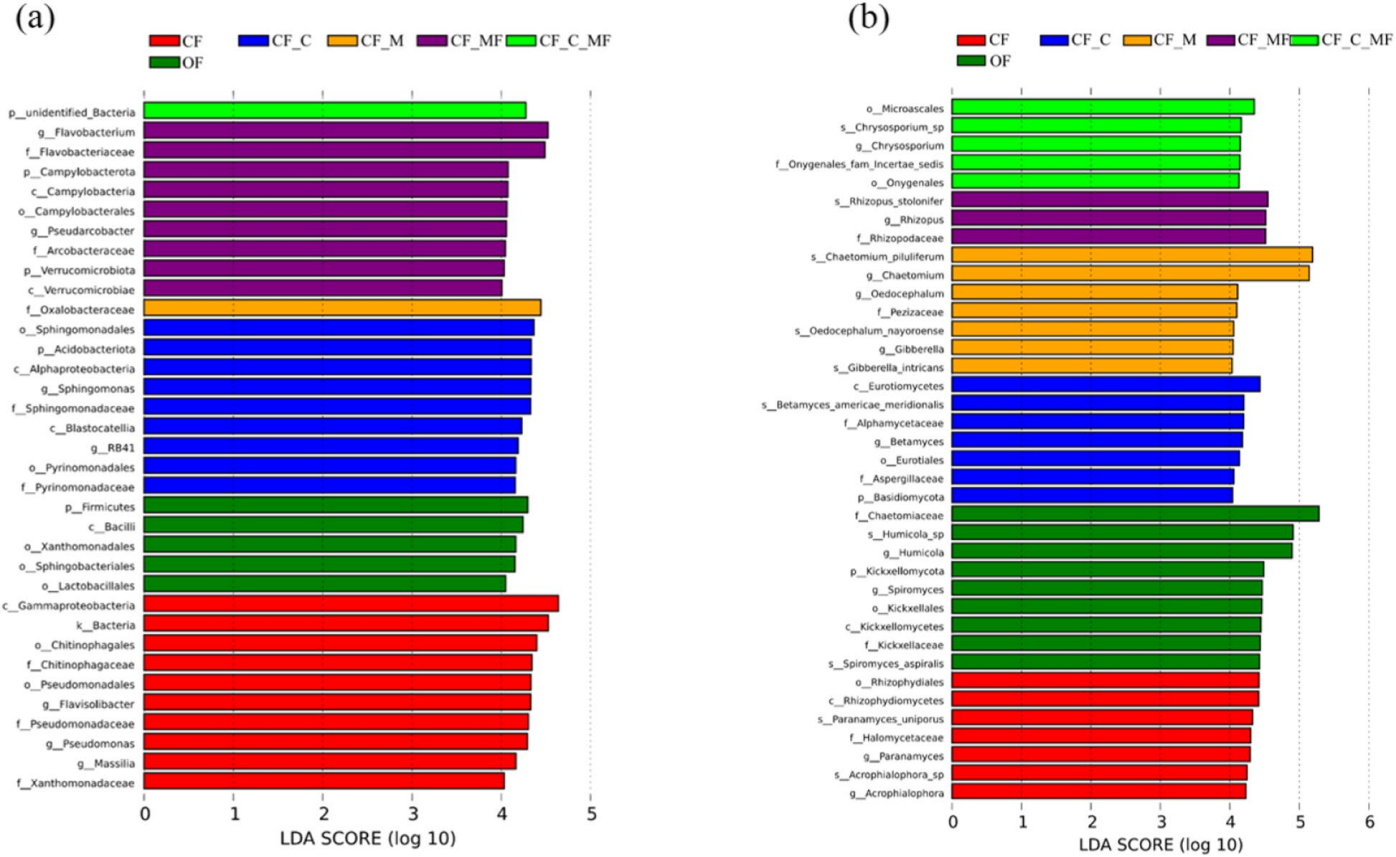
Line Discriminant Analysis Effect Size (LEfSe) analysis of the bacterial (**a**) and fungal (**b**) abundance for the soil with different soil treatments. Only the taxa meeting an LDA significance threshold of > 4 is shown. The species with significant differences in different groups in different groups, the length of the column diagram represents the influence of different species (that is, LDA Score). The letters p, c, o, f, g, and s represent the abbreviations for phylum, class, order, family, genus, and species, respectively

The linear discriminant analysis (LDA) effect size analysis determined statistically significant differences in taxon abundance. To further identify key bacterial or fungal taxa influencing the soil microbial community in lily fields under different soil treatments, a filtering criterion was set at an LDA value > 4. LEfSe (LDA Effect Size) analysis was applied to the sequencing results of six experimental groups. The LEfSe analysis highlights biomarkers significantly impacting the microbial community composition differences among treatment groups. These biomarkers are represented in the form of histograms with different colors in the LDA distribution plot. The length of the histograms represents the influence of different species. As shown in Fig. 5a, LEfSe analysis of bacteria showed that a total of 35 different species were obtained from different treatments. All treatments were significantly different at LDA ≥ 4 level, in the CF treatment, *Flavisolibacter*, *Pseudomonas*, and *Massilia* were significantly different; in the OF treatment, Firmicutes were significantly different; in the CF_C treatment, Acidobacteriota, *Sphingomonas*, and *RB41* were significantly different; in the CF_M treatment, no significant differences at phylum and genus level; in the CF_MF treatment, Campylobacterota, Verrucomicrobiota, *Flavobacterium*, and *Pseudarcobacter* were significantly different; in the CF_C_MF treatment, unidentified_Bacteria were significantly different. As shown in Fig. 5b, LEfSe analysis of the fungal showed that a total of 38 different species were obtained from the different treatments; in the CF treatment, *Paramyces* were significantly different; in the OF treatment, Kickxellomycota, *Humicola,* and *Spiromyces* were significantly different; in the CF_C treatment, Basidiomycota was significantly different; in the CF_M treatment, the *Chaetomium* and *Oedocephalum* were significantly different; in the CF_MF treatment, *Rhizopus* were significantly different; in the CF_C_MF treatment, there were no significant differences at the phylum and genus level.

### Multivariate analysis of soil microbial community structure and soil physicochemical properties

**Fig. 6:**
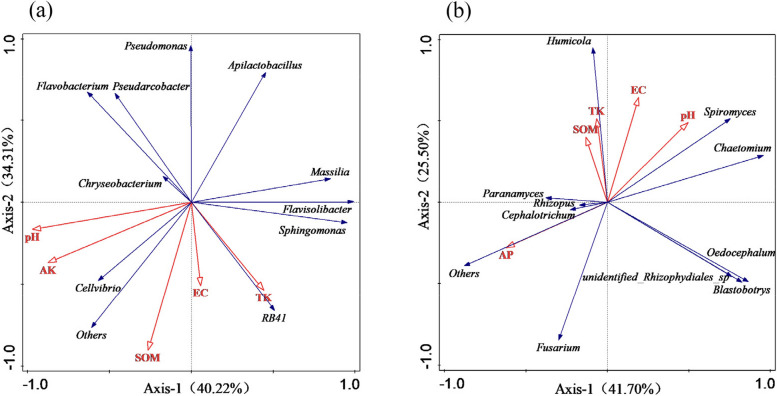
Redundancy analysis (RDA) of microbial communities with environmental variables based on bacterial genera (**a**) and fungal genera (**b**)

Redundancy analysis (RDA) indicated the influences of different soil physicochemical properties on microbial communities. As shown in Fig. 6a, for bacteria, the first two axes of RDA explained 40.22% and 34.31% of the total variation, respectively, cumulatively explaining 74.53% of the total variation. Among the various soil physicochemical properties, the pH ray was the longest, followed by organic matter, and it can be seen that pH and organic matter had the highest degree of influence on the soil bacterial community. *Cellvibrio* was positively correlated with pH and organic matter, and *RB41* was positively correlated with SOM and negatively correlated with pH. The pH and AK positively affected *Flavobacterium* and *Pseudarcobacter*.

As shown in Fig. 6b, for fungal, the first two axes of RDA explained 41.70% and 25.50% of the total variance, respectively, cumulatively explaining 67.21% of the total variance. The pH, EC, TK, and SOM had a positive effect on *Humicola*, *Spiromyces*, and *Chaetomium*.

### Self-toxic substances in different treated soils

Self-toxic substances are one of the important factors leading to continuous cropping obstacles. Compounds from root exudation and the decomposition of residual branches and fallen leaves may accumulate in the soil, potentially contributing to autotoxicity in plants [14, 15]. We use the GC–MS to identify self-toxic substances in different treatment soils, As shown in Table 2, it mainly includes 2,4-Di-*tert*-butylphenol (2,4-DTBP), esters, alcohols, ketones, hydrocarbons, and their derivatives. A total of fourteen organic compounds were detected in CF treatment, five organic compounds were detected in OF treatment, two organic compounds were detected in CF_C treatment, three organic compounds were detected in CF_M treatment, seven organic compounds were detected in CF_MF treatment, and three organic compounds were detected in CF_C_MF detected treatment. Autotoxins can affect the growth and development of medicinal plants by disrupting cell structures [16]. The decomposition of dead branches and fallen leaves produces secondary metabolites that can influence natural regeneration stages such as seed germination and seedling growth. Alkanes are compounds unique to the natural decomposition of dead branches and fallen leaves [15]. 2,4-Di-*tert*-butylphenol (2,4-DTBP) is the main self-toxic substance of root exudates, which directly and significantly inhibits the growth of Lanzhou lily [17]. The results showed that 2,4-DTBP existed in compound fertilizer treatment, and mixed carbon nanotubes treatments were not detected. Mix carbon nanotubes treatments detected the least non-toxic substances.

**Table 2:** The main organic compounds in six different treatments

Treatments	self-toxic substance components	molecular formula	CAS number
CF	2,4-Di-*tert*-butylphenol	C_14_H_22_O	96–76-4
	2,6,10-Trimethyldodecane	C_15_H_32_	3891–98-3
	3,8-Dimethylundecane	C_13_H_28_	17,301–30-3
	3-Methyl-5-Propylnonane	C_13_H_28_	31,081–18-2
	Tetracosane	C_24_H_50_	646–31-1
	2,6,11-Trimethyldodecane	C_15_H_32_	31,295–56-4
	3,7-Dimethylundecane	C_13_H_28_	17,301–29-0
	1,4-Benzenedicarboxylic acid, bis(2-ethylhexyl) ester	C_24_H_38_O_4_	6422–86-2
	Germacrane	C_15_H_30_	645–10-3
	1-Iodotridecane	C_13_H_27_I	35,599–77-0
	4,8-Dimethylundecane	C_13_H_28_	17,301–33-6
	1,1,2,2-Tetrachloroethane	C_2_H_2_Cl_4_	79–34-5
	Hexadecane	C_16_H_34_	544–76-3
	Heptacosane	C_27_H_56_	593–49-7
OF	1,2-dichloroethylbenzene	C_8_H_8_Cl_2_	1074–11-9
	1,4-Benzenedicarboxylic acid, bis(2-ethylhexyl) ester	C_24_H_38_O_4_	6422–86-2
	1-Iodododecane	C_12_H_25_I	4292–19-7
	2,7,10-Trimethyldodecane	C_15_H_32_	74,645–98-0
	Anthracene	C_14_H_30_	7225–67-4
CF_C	1,4-Benzenedicarboxylic acid, bis(2-ethylhexyl) ester	C_24_H_38_O_4_	6422–86-2
	2-Methoxyfurane	C_5_H_6_O_2_	25,414–22-6
CF_M	1,4-Benzenedicarboxylic acid, bis(2-ethylhexyl) ester	C_24_H_38_O_4_	6422–86-2
	5,5-Dimethylundecane	C_13_H_28_	17,312–73-1
	1,1,2,3-Tetrachloropropane	C_3_H_4_Cl_4_	18,495–30-2
CF_MF	1,4-Benzenedicarboxylic acid, bis(2-ethylhexyl) ester	C_24_H_38_O_4_	6422–86-2
	1-Dodecanol	C_12_H_26_O	112–53-8
	3-Methylundecane	C_12_H_26_	1002–43-3
	2,3,5-Trimethyldecane	C_13_H_28_	62,238–11-3
	2,5-Heptanedione	C_7_H_12_O_2_	1703–51-1
	1-Decanol	C_10_H_22_O	112–30-1
	1-Iodododecane	C_12_H_25_I	4292–19-7
CF_C_MF	1,4-Benzenedicarboxylic acid, bis(2-ethylhexyl) ester	C_24_H_38_O_4_	6422–86-2
	2-Ethyl-1-decanol	C_12_H_26_O	21,078–65-9
	5-Aminotetrazole	CH_3_N_5_	4418–61-5

## Discussion

Long-term continuous work will cause soil acidification and lack of soil nutrients [18]. Our research results show that the addition of mushroom residues treatment and organic fertilizer treatment can effectively alleviate the problem of soil acidification. Carbon nanotubes and mushroom residues flooded film have improved soil organic matter content, and soil elements, such as total phosphorus and available potassium. Soil health status affects crop growth and development, conductivity is one of the important factors, the higher the conductivity, the higher the concentration of soluble salt ions in the soil, which can harm plants and reduce yield and quality. The carbon nanotubes and mushroom residues flooded film significantly reduced soil electrical conductivity may be an important reason to alleviate the obstacles of continuous cropping.

After continuous cropping of medicinal plants, plant growth and development were inhibited and yield was reduced or even extinct. According to the plant height measurement, it can be seen that the plant height of mushroom residues flooded cover film treatment was significantly higher than other treatments, suggesting that the treatment could alleviate the inhibitory effect of the continuous crop barrier on the growth of lily plants, followed by the carbon nanotubes treatment, so the carbon nanotubes could promote growth, this was also verified in the study of Singh et al. [19]. Hu et al. [20] explored the molecular mechanisms by which multi-walled carbon nanotubes MWCNTs act on plants and found that they can improve plant growth by regulating key enzymes involved in carbon and nitrogen metabolism, which leads to increased carbohydrate production and nitrogen utilization and improved plant growth. The treatment of CF_M has a lower plant height compared to CF and OF treatments, possibly indicating a slower mineralization rate in this treatment. The nutrient levels in the soil may not be sufficient to meet the growth requirements of lilies.

With the development of molecular ecology research technology, Illumina NovaSeq sequencing technology has become an important way to study soil microorganisms and the characteristics of community structure [21], in this study, we analyzed soil microbial community structure, diversity indices, and species differences among six different soil treatments using Illumina NovaSeq sequencing technology, the results showed that organic fertilizer and carbon nanotubes and mushroom residues flooded film treatment significantly increased the diversity of bacterial communities. At the phylum level, Proteobacteria and Bacteroidota were the dominant phylum in all six treatments, with their proportion being the highest, constituting over 60% of the total bacterial abundance, followed by Acidobacteriota, Firmicutes, Verrucomicrobiota, and Gemmatimonadetes (Fig. 4a). The study of Lu et al. also indicates that the dominant phyla in continuous cropping lilies include Proteobacteria, Acidobacteria, Bacteroidetes, Actinobacteria, Chloroflexi and Gemmatimonadetes [22]. Wang et al. [23] found that microbial diversity in the rhizosphere soil of *Notopterygium incisum* was dominated by Proteobacteria and Bacteroidota, followed by Actinomycetes, Acidobacteriota, Gemmatimonadetes and Firmicutes. Li et al. [24] found Proteobacteria, Acidobacteriota, and Bacteroidota as the major bacterial taxa in soil samples of continuously cropped Andrographis paniculate. Pang et al. [25] found that the dominant bacterial phylum in sugarcane in different years of cropping were Proteobacteria, Actinomycetes, Acidobacteriota, Bacteroidota, Chloroflexi, Gemmatimonadetes, Cyanobacteria and Firmicutes. The above studies showed differences in the microbial dominant flora of different plant soil environments and similarities in the composition of the microbial communities of lily soil and soil in general. Compared with compound fertilizer treatment (Fig. 4a), Organic fertilizer and mushroom residues treatments reduced the relative abundance of Proteobacteria and Gemmatimonadetes. Carbon nanotubes treatment increased the relative abundance of Acidobacteriota by approximately 3.96%. Mushroom residues flooded film treatment increased the relative abundance of Verrucomicrobiota and Campylobacterota by 1.62% and 1.79% while decreasing the relative abundance of Firmicutes by 1.84%. Carbon nanotubes and mushroom residues flooded film treatment treatment increased the relative abundance of Acidobacteriota, unidentified_Bacteria, Verrucomicrobiota, Actinomycetes, and Chloroflexi by 2.40%, 3.81%, 1.39%, 0.80%, and 0.53%, respectively. Simultaneously, it decreased the relative abundance of Firmicutes by 2.56%.

Verrucomicrobiota is an important raw material for preparing ecological and environmentally friendly bacteria fertilizers and biological pesticides. It can promote the solubility of minerals through plant-microorganism interaction to increase soil mineral nutrition [26]. Actinobacteria is an important part of the soil ecosystem. The various secondary metabolic products produced have an important regulating role in other creatures in the soil, stimulating crop growth, assisting in the fixation of nitrogen, and controlling diseases and insect pests [27].

At the genus level, *Flavisolibacter*, *Sphingomonas*, *RB41*, and *Massilia* were the dominant genera shared by the six treatments. *Flavobacterium* is a gram-negative, aerobic or partially anaerobic rod-shaped bacterium [28], *Flavobacterium* is one of the most abundant taxa in the soil, especially in the rhizosphere [29], which can synthesize plant growth hormones as well as bioactive compounds against plant pathogens [30].

The results of the soil fungal diversity analysis showed that the six soil treatments altered the species composition of the soil fungal community to some extent, with little difference in fungal diversity among the treatments. At the phylum level, Ascomycota was the dominant phylum shared by the six treatments, accounting for approximately more than 50% of the total fungal abundance, followed by Chytridiomycota. Ascomycota has a wide adaptability, it is a class of saprophytic fungal, that can break down some difficult substances in the soil, and play an important role in the nutrition cycle [31, 32], it is relatively abundant in the soil of organic fertilizer and mushroom residues. Carbon nanotubes and mushroom residues flooded film treatment increases the relative abundance of Basidiomycota, Aphelidiomycota, and Mortierellomycota. The treatment of mushroom residues flooded film increases the relative abundance of Mucoromycota.

At the genus level, the relative abundance of each dominant bacterial genus did not differ much between treatments, and the dominant genera shared by the six treatments were *Chaetomium*, *Fusarium*, and *Humicola*. *Paranamyces* belonged to the Chytridiomycota, which was significantly increased in the compound fertilizer treatments and carbon nanotubes treatments.

Continuous cropping exacerbates the accumulation of soil self-toxic substances in plants, affects normal plant metabolism and growth, alters soil microbiology, and severely reduces their yield and quality [33]. The self-toxic substances in the root exudates can promote the occurrence of soil diseases to affect the plant [34]. When *Panax notoginseng* is continuously cultivated, some ginsenosides can accumulate in rhizosphere soils through root exudates or root decomposition, which hinders the emergence and growth of seedlings [35]. Phenolic acids are a self-toxic substance that promotes the growth of pathogenic fungi [36]. *Panax notoginseng* root exudates benzoic acid, phthalic acid, palmitic acid, and stearic acid have significant effects on the growth of *Panax notoginseng* roots, and activated charcoal has the effect of adsorbing chemosensory substances [37]. Carbon nanotubes are effective adsorbents for a wide range of organic compounds [38]. The relatively small variety of organic compounds detected by GC–MS in the treatment of carbon nanotubes and mushroom residues verified to some extent that carbon nanotubes can adsorb self-toxic substances.

## Conclusions

In conclusion, adding mushroom residues and organic fertilizers to the soil can alleviate soil acidification, and the sterilized environment created by the mushroom residues flooded film has a positive effect on soil physicochemical properties. Carbon nanotubes and mushroom residues flooded film treatment significantly increased microbial diversity and drove a shift in soil microbial community composition towards an increase in beneficial microflora, promoting plant growth, and the opportunity to adsorb self-toxic substances.

## Supplementary Information

**Supplementary Material 1.**

## Data Availability

The Data will be made available on reasonable request.
