# Supplementary material for: Reductive soil disinfestation by mixing carbon nanotubes and mushroom residues to mitigate the continuous cropping obstacles for Lilium Brownii
Source: Crop Health. 2024 Mar 15;2(1):3. doi: 10.1007/s44297-024-00023-2 (PMC12825970; doi:10.1007/s44297-024-00023-2)
Supplement: Supplementary file 1 — Supplementary Material 1. [file 44297_2024_23_MOESM1_ESM.pdf]

Supplementary Information

Reductive soil disinfestation by mixing carbon nanotubes and mushroom residues to mitigate the continuous cropping obstacles for *Lilium Brownii*

Ding-Di Tu[1], Rong Song[2], Bei Yan[1]*, Jin-Feng Dai[3], Hua Fang[4], Qian-Qi Zheng[1], Yi Gu[1], Xiao-Lan Shao[1], Hong Chen[1], Meng-Long Li[1], and Kai-Lin Liu[1]*

[1] College of Plant Protection, Hunan Agricultural University, Changsha 410128, PR China

[2] Institute of Agricultural Environment and Ecology, Hunan Academy of Agricultural Sciences, Changsha, 410125, China

[3] Hunan Provincial Institute of Product and Goods Quality Inspection, Changsha 410007, China

[4] Key Laboratory of Molecular Biology of Crop Pathogens and Insects, Ministry of Agriculture, Zhejiang Provincial Key Laboratory of Biology of Crop Pathogens and Insects, Institute of Pesticide and Environmental Toxicology, College of Agriculture and Biotechnology, Zhejiang University, Hangzhou 310058, China

* Corresponding author: kailin@hunau.net (Kai-Lin Liu), 670561845@qq.com (Bei Yan)

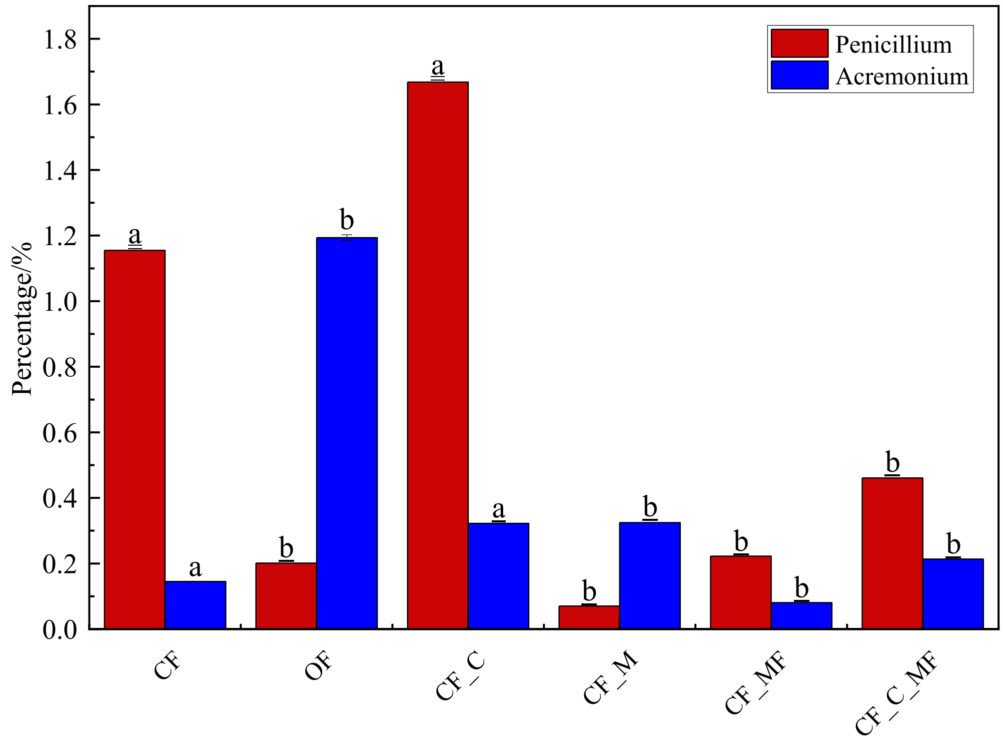

Fig. S1 The percentage changes of two major pathogenic bacteria in each treatment group. Different letters indicate significant differences between the groups (P<0.05).
